# Cheek cell fatty acids reflect *n*-3 PUFA in blood fractions during linseed oil supplementation: a controlled human intervention study

**DOI:** 10.1186/1476-511X-12-173

**Published:** 2013-11-14

**Authors:** Annemarie Grindel, Frank Staps, Katrin Kuhnt

**Affiliations:** 1 Department of Nutritional Physiology, Institute of Nutrition, Friedrich Schiller University, Dornburger Straße 24, Jena 07743, Germany

**Keywords:** Oral mucosa, Alpha-linolenic acid, Long-chain *n*-3 PUFA, Buccal cells, Fatty acid supplementation, Plasma, Red blood cells, Peripheral blood mononuclear cells, Olive oil

## Abstract

**Background:**

Adequate biomarkers for the dietary supply of fatty acids (FA) are FA of adipose tissue and blood fractions. In human studies, invasive sample collection is unpleasant for subjects. In contrast, cheek cell sampling can be considered as a non-invasive alternative to investigate FA status.

The aim of this study was to analyze whether cheek cell FA composition reflect the supplementation of alpha-linolenic acid (ALA) using a linseed oil mixture compared to olive oil supplementation. Additionally, it was investigated if cheek cell FA composition correlates with the FA composition of plasma, red blood cells (RBC) and peripheral blood mononuclear cells (PBMC) before and during both interventions.

**Methods:**

During a 10-week randomized, controlled, double-blind human intervention study, 38 subjects provided cheek cell and blood samples. After a two-week run-in period, the test group (n = 23) received 17 g/d of an ALA-rich linseed oil mixture, while the control group (n = 15) received 17 g/d of an omega-3 (*n*-3) polyunsaturated FA (PUFA)-free olive oil. Cheek cells and blood were collected on days 0, 7 and 56 of the 8-week intervention period.

**Results:**

Compared to olive oil, the linseed oil intervention increased ALA and also the endogenously converted long-chain *n*-3 metabolites eicosatetraenoic-, eicosapentaenoic- and docosapentaenoic acid in cheek cells (*P ≤ 0.05*). Docosahexaenoic acid remained unchanged. Reflecting the treatment, the *n*-6/*n-*3 ratio decreased in the test group. In general, cheek cell FA reflected the changes of FA in blood fractions. Independent of treatment, significant correlations (*P ≤ 0.05*) of *n*-6 PUFA and *n-*3 PUFA between cheek cells and plasma, RBC and PBMC were found, except for linoleic acid and ALA.

**Conclusions:**

The changes in FA composition of cheek cells confirmed that ALA from linseed oil increased endogenously derived *n-*3 PUFA in cheek cell lipids. These changes in cheek cells and their correlation to the respective FA in blood fractions indicate the cheek cell FA profile as an adequate non-invasive biomarker for short-term *n*-3 PUFA intake and metabolism. Therefore, cheek cell FA can be used in human intervention studies or large-scale epidemiological studies, especially for assessment of the *n*-3 PUFA status.

**Trial registration:**

ClinicalTrials.gov, IDNCT01317290

## Background

Based on ongoing discussions about the effect of dietary fat quality on chronic diseases including cardiovascular diseases (CVD) [1], it is of increasing interest to qualitatively and quantitatively assess dietary intake of fatty acids (FA). A special focus is on omega-3 (*n*-3) polyunsaturated FA (PUFA) intake and their enrichment in the body. There have been numerous reports on the physiologically beneficial effects of *n*-3 PUFA, including a reduction of CVD risk [1,2], anti-inflammatory effects [1,3] and associations with mental health [4,5]. A desire exists to find an adequate biomarker that can reflect the FA intake and FA status in humans [6]. However, a satisfying gold standard biomarker that adequately reflects FA intake and status does not exist. Food frequency questionnaires as used in large-scale population studies only allow speculations on the dietary FA intake but not on the enrichment of FA in the body pool. The *n*-3 index which can be determined from red blood cells (RBC) is considered a biomarker, or even a risk factor, for CVD [7]. However, the collection procedure for measurement of FA in RBC or other biomarkers of choice as FA of whole blood, plasma, blood cells, and adipose tissue [8,9], is invasive and an additional burden to subjects in human intervention studies.

To overcome such problems, the use of cheek cells as an alternative material reflecting dietary FA composition has been proposed [10,11]. In that context, McMurchie *et al.* were the first to use cheek cell FA for the assessment of the FA composition in humans [12]. Cheek cells have a rapid and constant turnover and show a fast regeneration time of approximately 5 days [13]. Therefore, cheek cell FA are thought to reflect short-term changes in the diet [11]. Cheek cells can be obtained by a mouthwash or by scraping the inside of a cheek, e.g. with a brush [11,12]. Along with its non-invasive nature, utilizing cheek cells is cost effective and can be applied in a non-clinical environment without medicinal personal on a large scale [14]. This method is especially advantageous when used with infants or children, as they are often afraid of blood sampling. Moreover, this procedure might also be an improvement for the elderly, as blood removal can be difficult due to inaccessible veins. However, despite its convenience and applicability, the use of cheek cell FA as a biomarker for FA intake and *n*-3 PUFA status has rarely been used in human intervention studies and is not yet fully established. One reason could be the small sample amount of cheek cells obtained, which requires precise sampling procedures for sufficient cell amounts [11,14].

To validate the method’s significance, the aim of the present study was to detect the FA composition of cheek cells with focus on *n*-3 PUFA without intervention and during supplementation with either alpha-linolenic acid (ALA)-rich linseed oil mixture or with *n*-3 PUFA-free olive oil. Additionally, it was of interest if the cheek cell FA composition correlates to the FA composition of plasma, RBC and peripheral blood mononuclear cells (PBMC).

## Study design and methods

The double-blind, parallel arm, randomized, controlled study was approved by the ethics committee of the Friedrich Schiller University of Jena (No. 2270-04/08) and is registered in Clinical Trials (NCT01317290). The implementation of the study and all sample analyses were carried out at the Department of Nutritional Physiology of the Friedrich Schiller University Jena, Germany.

### Subjects

The present cheek cell study was part of a main study. In the main project a total of 77 subjects were recruited for the entire study from the general population by advertisement. Participants were randomly allocated to a test group with linseed oil intervention and a control group with olive oil intervention using computer-generated random numbers according to age, gender, and BMI. Exclusion criteria were pregnancy, lactation, lactose intolerance, chronic diseases, and medication influencing fat metabolism or inflammation.

From the total study population 43 healthy subjects with BMI < 25 (23 subjects of the test group and 20 subjects of the control group) were willing to donate cheek cell samples in addition to blood samples and therefore built the cheek cell subgroup. Unfortunately, baseline values of 5 control subjects were not available, thus the cheek cell subgroup was created by 23 participants (13 males, 10 females) receiving the test oil and only 15 participants (6 males, 9 females) receiving the control oil (Table 1). Before enrollment into the study, all subjects were required to sign a declaration of consent. A baseline questionnaire about health, lifestyle and nutritional habits was completed by each participant. At the end of the study, an anonymous compliance protocol of the study regime was completed by the subjects. In addition, compliance was measured by counting used oil packets.

**Table 1:** Baseline characteristics of study participants

	**Test group**		**Control group**		
	**Linseed oil mix (n = 23)**		**Olive oil (n = 15)**		
	**Mean ± SD**	**Median**	**Mean ± SD**	**Median**	***P***
Age [years]	28.1 ± 11.1	25.0	38.1 ± 17.9	25.0	0.180^1^
Body weight [kg]	66.2 ± 9.9	66.3	66.4 ± 11.8	70.9	0.961^2^
Body mass index [kg/m^2^]	22.2 ± 2.2	22.0	22.6 ± 2.8	22.2	0.599^2^
Waist circumference [cm]	81.2 ± 5.0	81.0	82.3 ± 9.7	83.0	0.692^2^
Blood pressure systolic [mmHg]	134 ± 17.3	133.0	134 ± 14.1	134.0	0.869^2^
Blood pressure diastolic [mmHg]	82.9 ± 6.7	82.0	85.3 ± 7.1	85.0	0.240^2^

Differences between the test group and control group: ^1^*P* value according to Mann Whitney U test, ^2^*P* value according to student *t* test.

### Study design and supplements

The duration of the study was 10 weeks, separated into two periods: a two-week run-in period and an eight-week intervention period. During the entire study, subjects were not allowed to consume fish, fish-oil, *n*-3 PUFA-rich oils and linseeds. A three-day standardized diet was provided to the subjects in the last days of each study period. No other food consumption was intended during the standardized diet [15]. Blood and cheek cell sampling was conducted at the end of the run-in period (day 0), after one week of intervention (day 7) and at the end of the intervention (day 56).

During the two-week run-in period, 17 g of *n*-3 PUFA-free run-in oil mixture with a FA composition of an average Western diet was given as a supplement (Table 2). The run-in oil was provided as a spread and consisted of different fats and oils (coconut fat, palm oil, palm kernel fat, olive oil, sunflower oil, and chocolate cream). The test oil mainly contained linseed oil (Erfurter Ölmühle, Germany) mixed with sunflower oil (K-Classic, Germany) to receive a ratio of linoleic acid (LA)/ALA of 0.7/1.0. The composition of the test oil was characterized by a high content of total PUFA (>50%) consisting of 31% ALA and 22% LA (Table 2). The control oil was a highly refined olive oil (Gustav Heess GmbH, Germany) with a small amount of PUFA (6%), mainly LA, and a high amount of the monounsaturated FA (MUFA) oleic acid (OA) at 78% (Table 2). According to gender specific energy recommendations, female subjects consumed 15.5 g/d and male subjects 18.5 g/d of the test oil or control oil during the intervention period. The oil was mainly taken pure or in addition to other food. Subjects were instructed to store the oils in the cold (4-7°C) and dark. Participants and scientific staff involved in the study were blinded. Oil cups of test and control oil were standardized and labeled using a numeric code.

**Table 2:** FA composition of supplemented oils and daily consumed dose during the study

	**Run-in oil**		**Linseed oil mix**		**Olive oil**	
	**Total subjects (n = 38)**		**Test group (n = 23)**		**Control group (n = 15)**	
	**Oil**	**Intake**^**1**^	**Oil**	**Intake**^**1**^	**Oil**	**Intake**^**1**^
**Major FA**	**[% FAME]**	**[g/d]**	**[% FAME]**	**[g/d]**	**[% FAME]**	**[g/d]**
*Daily dose*		17.0		17.0		17.0
Σ SFA	36.6	5.60	8.99	1.38	13.9	2.13
C18:1*n*-9 (OA)	45.1	6.90	36.3	5.55	77.5	11.9
C18:2*n*-6 (LA)	16.8	2.60	21.6	3.30	5.52	0.85
C18:3*n*-3 (ALA)	0.18	0.03	31.1	4.85	0.52	0.08
Σ*n*-6/Σ*n*-3	94.0/1.0		0.7/1.0		11.0/1.0	

FA of supplemented oils are presented as means [% FAME].

^1^Daily intake is given as mean of men and women; men received 18.5 g/d and women 15.5 g/d total daily dose; based on average 2800 kcal for men and 2200 kcal for women to achieve comparable energy% of the supplemented FA for both sexes.

### Cheek cell sampling

Cheek cell sampling was performed in the morning after the fasting blood samples were taken. The subjects were instructed not to eat or drink, but to brush their teeth prior to cheek cell sampling. Before cell collection, subjects had to clean their mouth by rinsing with 20 ml distilled water. The cheek cell removal was performed with sterile cell brushes (Cytobrush® Plus GT, Medscand® Medical, Germany) by scraping and rotating one brush up and down (20 times) on the inside of each cheek. Both cell brushes were placed in 50 ml tubes containing 10 ml distilled water and covered with a screw cap. Subsequently, subjects rinsed their mouth again with 20 ml distilled water which was then added to the 50 ml tube. The tubes were stored on ice. Cheek cells were suspended in the water in the tube and each brush was rinsed with 2.5 ml distilled water to remove the majority of the cells. After centrifugation (10 min, 3750 × g) the supernatant was carefully discarded. The cell pellets were washed once again with distilled water, and then suspended in a total volume of 4 ml distilled water for transfer into a 4 ml tube. The samples were stored at -80°C until the end of the study (approx. 4 months).

For analyzing the distribution of lipid fractions of total cheek cell lipids and phospholipids (PL), separate independent cheek cell samples were obtained and pooled (n = 10). Fractionation was done by use of high performance thin-layer chromatography and scanned by means of densitometry at 400 nm [16].

### Fatty acid analysis of cheek cells, plasma, RBC, PBMC

The blood sampling, the preparation of plasma, RBC and PBMC and the GC method were performed according to Kuhnt *et al.*[15,17]. Lipid extraction of cheek cell and blood samples was conducted with chloroform/methanol/water (2:1:1; v/v/v) according to Folch *et al*. [18]. The transesterification was performed with boron triflouride. The purification of fatty acid methyl esters (FAME) by thin-layer chromatography, e. g. exclusion of the cholesterol fraction was only conducted for blood fractions. In cases of the cheek cell samples purification was omitted caused by small sample amounts. FAME were analyzed by gas chromatography with flame ionization detector (GC-17 V3, Shimadzu, Japan; column: DB-225MS, 60 m × 0.25 mm i.d. with 0.25 μm film thickness, Agilent Technologies, U.S.). Oven temperature was initially maintained at 70°C for 2 min, increased by 10°C/min to 180°C, further increased by 2°C/min to 220°C and held for 5 min. During the final step it was increased by 2°C/min to 230°C and held for 27 min.

The injection volume for blood fraction extracts was 1 μl and for cheek cell lipid extracts was 2 μl with a split mode of 1:20, respectively. In all analyzed materials the same 47 fatty acids were integrated (C10 - C24). Individual FAME were expressed as a percentage of total identified FAME peak areas [% of total FAME; % FAME]. A series of external standards were used for the qualitative analysis of FAME (No.463, 674 (Nu-Check Prep, USA), BR2, BR4, ME93 (Larodan, Sweden), Supelco®37 Component FAME Mix (Supelco, USA) and PUFA No.3 (Matreya LLC, USA). The peak integration of the chromatograms was performed with LabSolutions software for gas-chromatography (GCsolution, Shimadzu, Japan).

### Statistical analyses

All statistical analyses were performed using IBM SPSS software, version 20.0 (IBM, USA) with *P ≤ 0.05* indicating significance. FAME levels are given as mean and standard deviation (SD). Data were tested for normal distribution and variance homogeneity. The *t*-test was used for comparison of the two treatment groups. When data were not normally distributed according Kolmogorov Smirnov test the comparison was based on the Mann Whitney U Test.

Effects on FA composition within each treatment on FA composition were analyzed with general linear model repeated-measures analysis. To test the differences at the end of the study (day 56) between the treatments (treatment as fixed factor) baseline values and age were used as covariate (univariate ANCOVA) using bonferroni adjustment. FA portions of cheek cells were compared with FA portions of different blood fractions using one-way ANOVA with Dunnett-T3 test for multiple comparison. A correlation analysis was conducted between FA of cheek cells, plasma, RBC and PBMC using Pearson’s correlation analysis.

## Results

### Baseline characteristics

The baseline body weight, body mass index (BMI), waist circumference and blood pressure did not differ between the test group and control group (Table 1). The age distribution between the study groups differed, but had similar median age of 25 years. All 38 subjects of this cheek cell subgroup finished the entire study successfully and consumed the supplemented oils (by self-report and counted oil packages).

### Cheek cell FA composition

The analysis of the lipid fractions of cheek cell lipids was conducted independent of cheek cell samples of the study. Therefore, the presented data refer to pooled cheek cell samples of various donors. Cheek cell lipids mainly contained PL (57%), whilst triglycerides, cholesterol and non-esterified FA amounted to 20%, 19% and 4%, respectively. Moreover, the total PL fraction comprised of similar proportions of phosphatidylcholine, sphingomyelin and phosphatidylethanolamine (35%, 31%, 30% of total PL), whereas only 3% phosphatidylinositol was present. Lastly, lysophosphatidylcholine and phosphatidylserine were below the detection limit.

Regarding cheek cell samples of the intervention study, the main FA portions at baseline were the saturated fatty acids (SFA) palmitic acid (C16:0) and stearic acid (C18:0), the *n*-9 MUFA OA and the *n*-6 PUFA LA. Compared to these, *n*-3 PUFA portions only contribute less than 2% to the FA composition of cheek cells (Tables 3 and 4).

**Table 3:** Cheek cell FA composition during supplementation with linseed oil mix and olive oil

**FA [% FAME]**	**Test group - linseed oil mix (LO) (n = 23)**			**Control group - olive oil (OO) (n = 15)**			**LO x OO**
	**Day 0**	**Day 7**	**Day 56**	**Day 0**	**Day 7**	**Day 56**	**Day 56**
	**Mean ± SD**	**Mean ± SD**	**Mean ± SD**	**Mean ± SD**	**Mean ± SD**	**Mean ± SD**	***P***^**1**^
*SFA*							
C16:0	17.1 ± 2.20^a^	16.9 ± 2.14^ab^	15.9 ± 1.75^b^	17.0 ± 1.55	16.9 ± 1.89	16.6 ± 2.18	0.590
C18:0	16.7 ± 3.82	15.9 ± 3.08	15.8 ± 2.78	16.3 ± 2.71	15.3 ± 1.60	16.1 ± 2.25	0.672
*n-9 MUFA*							
C18:1*n*-9 (OA)	27.0 ± 3.76^b^	26.6 ± 3.00^b^	28.0 ± 2.93^a^	26.4 ± 2.47^b^	27.8 ± 1.75^a^	27.9 ± 2.59^a^	0.222
Total *n*-9	27.4 ± 3.78^b^	27.0 ± 3.01^b^	28.4 ± 2.92^a^	26.8 ± 2.49^b^	28.3 ± 1.76^a^	28.4 ± 2.55^a^	0.206
*n-6 PUFA*							
C18:2*n*-6 (LA)	16.6 ± 2.01^b^	18.2 ± 4.79^ab^	17.4 ± 1.93^a^	17.9 ± 2.04	17.8 ± 1.90	16.6 ± 1.61	0.070
C18:3*n*-6	0.10 ± 0.03^a^	0.09 ± 0.03^b^	0.10 ± 0.04^ab^	0.09 ± 0.03	0.10 ± 0.02	0.11 ± 0.03	0.306
C20:3*n*-6	1.20 ± 0.28	1.13 ± 0.33	1.18 ± 0.25	1.09 ± 0.13^b^	1.09 ± 0.22^b^	1.18 ± 0.23^a^	0.108
C20:4*n*-6 (AA)	3.15 ± 0.74	3.18 ± 0.89	3.30 ± 0.73	3.58 ± 0.74	3.61 ± 0.68	3.71 ± 0.69	0.215
C22:5*n-*6	0.06 ± 0.03	0.06 ± 0.03	0.07 ± 0.05	0.07 ± 0.01^b^	0.07 ± 0.02^b^	0.08 ± 0.02^a^	0.259
Total *n*-6	21.6 ± 2.51^b^	23.2 ± 4.70^ab^	22.6 ± 2.33^a^	23.4 ± 2.18	23.3 ± 1.94	22.3 ± 1.89	0.350
*n-3 PUFA*							
C18:3*n*-3 (ALA)	0.27 ± 0.10^c^	0.39 ± 0.09^b^	0.50 ± 0.18^a^	0.31 ± 0.18	0.25 ± 0.06	0.26 ± 0.11	0.001
C20:4*n*-3 (ETA)	0.06 ± 0.03^c^	0.08 ± 0.05^b^	0.11 ± 0.03^a^	0.05 ± 0.01	0.05 ± 0.02	0.05 ± 0.02	0.001
C20:5*n*-3 (EPA)	0.21 ± 0.08^b^	0.23 ± 0.09^b^	0.33 ± 0.11^a^	0.22 ± 0.08	0.24 ± 0.11	0.22 ± 0.07	0.001
C22:5*n*-3 (DPA)	0.20 ± 0.07^b^	0.20 ± 0.08^b^	0.30 ± 0.10^a^	0.27 ± 0.09	0.29 ± 0.10	0.26 ± 0.09	0.006
C22:6*n*-3 (DHA)	0.77 ± 0.24	0.83 ± 0.34	0.76 ± 0.28	0.82 ± 0.28	0.82 ± 0.29	0.76 ± 0.22	0.796
Total *n*-3	1.62 ± 0.37^c^	1.87 ± 0.50^b^	2.16 ± 0.58^a^	1.77 ± 0.51	1.75 ± 0.45	1.70 ± 0.42	0.001
*Sum*							
Σ SFA	40.8 ± 6.23^a^	39.6 ± 6.01^ab^	38.3 ± 4.69^b^	39.8 ± 3.31	38.8 ± 2.78	39.5 ± 4.48	0.616
Σ MUFA	35.9 ± 5.04	35.3 ± 3.92	36.8 ± 4.19	34.9 ± 2.65^b^	36.0 ± 2.31^a^	36.4 ± 3.23^a^	0.294
Σ PUFA	23.3 ± 2.72^b^	25.2 ± 4.65^b^	24.9 ± 2.66^a^	25.3 ± 2.46	25.2 ± 1.93	24.1 ± 1.85	0.099
*Ratio*							
*n*-6/*n*-3	13.9 ± 3.22^a^	13.5 ± 5.63^a^	11.0 ± 2.44^b^	13.9 ± 2.75	14.1 ± 3.63	13.9 ± 3.75	0.001
AA/EPA	15.9 ± 4.33^a^	14.8 ± 4.19^a^	10.6 ± 2.49^b^	17.5 ± 5.50	17.1 ± 5.64	18.2 ± 7.00	0.001

Values are presented as mean ± SD.

All FA were statistically analyzed with GLM repeated-measured analysis; ^abc^Different letters indicate significant differences between FA proportions within the treatment groups (*P* ≤ 0.05); ^1^Differences between the test group and control group at day 56 (end of intervention) were analyzed with GLM univariate ANCOVA (age and baseline value as covariate, pairwise comparison with bonferroni adjustment).

**Table 4:** Comparison of FA profile in cheek cells, plasma, RBC and PBMC (baseline; n = 38)

**FA [% FAME]**	**Cheek cells**	**Plasma**	**RBC**	**PBMC**
	**Mean ± SD**	**Mean ± SD**	**Mean ± SD**	**Mean ± SD**
*SFA*				
C16:0	17.1 ± 1.95^a^	23.9 ± 1.46^b^	28.2 ± 1.46^c^	19.3 ± 1.32^d^
C18:0	16.5 ± 3.39^a^	6.57 ± 0.73^b^	10.7 ± 1.14^c^	20.5 ± 1.16^d^
*n-9 MUFA*				
C18:1*n*-9 (OA)	26.7 ± 3.29 ^a^	20.9 ± 1.99 ^b^	17.2 ± 1.06^c^	16.3 ± 0.85^c^
Total *n*-9	27.2 ± 3.30^a^	21.2 ± 2.00^b^	17.6 ± 1.09^c^	17.2 ± 0.86^c^
*n-6 PUFA*				
C18:2*n*-6 (LA)	17.1 ± 2.10^a^	30.8 ± 3.15^b^	13.6 ± 1.14^c^	5.76 ± 1.51^d^
C18:3*n*-6	0.10 ± 0.03^a^	0.29 ± 0.09^b^	0.05 ± 0.01^c^	0.05 ± 0.01^c^
C20:3*n*-6	1.16 ± 0.24^a^	1.50 ± 0.31^b^	1.56 ± 0.31^b^	1.40 ± 0.30^b^
C20:4*n*-6 (AA)	3.32 ± 0.76^a^	6.39 ± 1.05^b^	13.9 ± 0.93^c^	24.8 ± 1.42^d^
C22:5*n-*6	0.06 ± 0.02^a^	0.12 ± 0.04^b^	0.38 ± 0.10^c^	0.24 ± 0.07^d^
Total *n*-6	22.3 ± 2.52^a^	39.4 ± 3.18^b^	32.7 ± 1.42^c^	34.8 ± 1.62^d^
*n-3 PUFA*				
C18:3*n*-3 (ALA)	0.29 ± 0.13^a^	0.40 ± 0.07^b^	0.14 ± 0.03^c^	0.06 ± 0.02^d^
C20:4*n*-3 (ETA)	0.05 ± 0.02^a^	0.07 ± 0.03^b^	0.06 ± 0.02^b^	0.04 ± 0.01^b^
C20:5*n*-3 (EPA)	0.22 ± 0.08^a^	0.51 ± 0.16^b^	0.75 ± 0.28^c^	0.31 ± 0.08^d^
C22:5*n*-3 (DPA)	0.22 ± 0.08^a^	0.39 ± 0.11^b^	2.09 ± 0.33^c^	1.56 ± 0.28^d^
C22:6*n*-3 (DHA)	0.79 ± 0.25^a^	1.44 ± 0.39^b^	3.91 ± 0.90^c^	1.66 ± 0.32^d^
Total *n*-3	1.68 ± 0.43^a^	3.06 ± 0.55^b^	7.07 ± 1.21^c^	3.69 ± 0.45^d^
*Sum*				
Σ SFA	40.4 ± 5.24^ac^	32.6 ± 1.25^b^	40.4 ± 0.89^a^	41.9 ± 1.27^c^
Σ MUFA	35.5 ± 4.24^a^	25.0 ± 2.53^b^	19.8 ± 1.19^c^	19.7 ± 0.94^c^
Σ PUFA	24.1 ± 2.77^a^	42.4 ± 3.20^b^	39.8 ± 1.11^c^	38.5 ± 1.42^d^
*Ratio*				
*n*-6/*n*-3	13.9 ± 3.01^a^	13.3 ± 2.79^a^	4.77 ± 0.94^b^	9.58 ± 1.45^c^
AA/EPA	16.6 ± 4.81^a^	13.7 ± 4.67^a^	20.5 ± 6.45^b^	85.4 ± 21.5^c^

Values are presented as Mean ± SD.

^abcd^Indicates significant differences between FA of the different matrices (one-way ANOVA; post-hoc Dunnett-T3 test for multiple comparison; *P* ≤ 0.05).

The linseed oil supplementation in the test group (n = 23) resulted in a significant increase of ALA portion in cheek cells from 0.27% FAME at baseline, to 0.39% FAME after 7 days and to 0.50% FAME after 56 days of intervention (*P ≤ 0.001*; Table 3). In addition, long-chain *n-*3 PUFA, such as eicosatetraenoic acid (ETA), eicosapentaenoic acid (EPA) and docosapentaenoic acid (DPA), endogenously derived from ALA as precursor and increased significantly in cheek cells after 56 days of linseed oil intervention. However, docosahexaenoic acid (DHA) portion remained unchanged. The *n*-3 PUFA ALA and ETA already showed significant increase after 7 days, while the conversion products EPA and DPA increased only after 56 days of supplementation. Due to the increase of *n*-3 PUFA in cheek cells caused by linseed oil intervention, the ratios of *n-*6/*n-*3 PUFA as well as arachidonic acid (AA)/EPA decreased significantly from 13.9 to 11.0 and from 15.9 to 10.6, respectively. Additionally, LA and total *n-*6 PUFA portions increased during linseed oil intervention (*P ≤ 0.009*). The olive oil supplementation in the control group (n = 15) resulted in a slight increase of OA in cheek cells from 26.4% FAME at baseline to 27.9% FAME after 56 days (*P ≤ 0.05*). No changes occurred in any *n*-3 PUFA in the control group (Table 3).

Given the fact that the FA composition of cheek cells in both groups did not differ at baseline; at the end of the study, a significantly higher portion of *n*-3 PUFA, such as ALA, ETA, EPA, DPA, and total *n*-3 PUFA, was detected in the test group as compared to the controls. The ratios of *n*-6/*n*-3 PUFA and AA/EPA were significantly lower in the test group compared to controls after intervention (Table 3).

In addition, a subgroup of seven subjects allocated to the test group provided cheek cells after one week of wash-out at day 63. Interestingly, a significant decrease of ALA (day 0: 0.24, day 56: 0.48, day 63: 0.28% FAME) and ETA portions (day 0: 0.05, day 56: 0.12, day 63: 0.07% FAME) and a slight but not significant decrease of EPA (day 0: 0.21, day 56: 0.38, day 63: 0.35% FAME) and DPA portions (day 0: 0.20, day 56: 0.32, day 63: 0.29% FAME) were already detectable one week post-supplementation.

### Comparison of FA composition of cheek cells with plasma, RBC and PBMC

Comparing the FA composition of cheek cells with plasma, RBC and PBMC, it should be considered that each fraction has its specific FA profile (Table 4). Therefore, partially strong variations occurred regarding the FA composition between the four fractions. Almost all analyzed FA of plasma, RBC, PBMC and cheek cells at baseline were significantly different (*P ≤ 0.05*) to each other (Table 4).

In general, cheek cells contained the lowest portion of total PUFA and the highest portion of OA and therefore of MUFA. Total *n*-3 PUFA portion of cheek cells amounted to only ~1.7% FAME. In contrast, total *n-*3 PUFA portion of RBC amounted for ~7.1% FAME and therefore was almost 4.5-fold higher than in cheek cells (Table 4). Especially DPA and DHA differed between the fractions and were therefore 2 - 8-fold higher in plasma and even 5 - 10-fold higher in RBC compared to cheek cells (Table 4).

After the intervention with linseed oil or olive oil, changes in lipids of the blood material (data not shown) were comparable to changes of cheek cell lipids. Upon closer examination, the *n*-3 PUFA ALA, ETA, EPA and DPA significantly increased in lipids of plasma, RBC and PBMC after linseed oil intervention, whereas *n*-3 PUFA remained unchanged in the olive oil group.

### Correlation between FA of cheek cells with FA of plasma, RBC and PBMC

No significant correlations were found between SFA of cheek cells and SFA of the three blood fractions at either baseline or during both treatments. At baseline, OA and total *n*-9 MUFA correlated between cheek cells and plasma (*r* 0.34 and 0.33, respectively), but not between cheek cells and RBC or PBMC (Table 5). Individual *n-*6 PUFA such as gamma-linolenic acid (C18:3*n*-6), dihomo-gamma-linolenic acid (C20:3*n*-6), AA, and DPA*n*-6 showed significant correlations between cheek cells and plasma (*r* 0.33, 0.41, 0.54 and 0.52, respectively) or RBC (*r* 0.44, 0.47, 0.54 and 0.58, respectively) (Table 5). However, no significant correlations between cheek cells and the blood fractions were found in LA and total *n*-6 PUFA at baseline. Interestingly, total *n*-6 PUFA and also total PUFA showed negative correlations (not significant) between cheek cells and the three blood fractions. In contrast, all analyzed *n*-3 PUFA of cheek cells correlated significantly with the respective *n*-3 PUFA in plasma, RBC and PBMC (except for ALA) (*r* 0.47 - 0.82; Table 5). Plasma lipids correlated significantly with RBC and PBMC lipids in regard of *n*-3 PUFA. In addition, single *n*-6 PUFA and single SFA correlated significantly between plasma and RBC lipids, while less correlation were found between PBMC and plasma lipids (Table 5).

**Table 5:** Correlation between cheek cell FA with FA of plasma, RBC and PBMC

	**Baseline**					**Intervention**^***‡***^					
	**Total subjects**					**Test group – linseed oil mix**			**Control group – olive oil**		
	**(n = 38)**					**(n = 46)**			**(n = 30)**		
	**Cheek cell correlation**			**Plasma correlation**		**Cheek cell correlation**			**Cheek cell correlation**		
	**Plasma**	**RBC**	**PBMC**	**RBC**	**PBMC**	**Plasma**	**RBC**	**PBMC**	**Plasma**	**RBC**	**PBMC**
	***r***^***1***^	***r***^***1***^	***r***^***1***^	***r***^***1***^	***r***^***1***^	***r***^***1***^	***r***^***1***^	***r***^***1***^	***r***^***1***^	***r***^***1***^	***r***^***1***^
*SFA*											
C16:0	-0.08	-0.1	0.09	0.80**	0.31	-0.01	-0.2	-0.1	0.34	-0.17	0.34
C18:0	0.04	-0.16	-0.07	0.60**	0.15	0.09	0.04	-0.14	0.05	0.22	-0.03
*n-9 MUFA*											
C18:1*n*-9 (OA)	0.34*	0.06	0.19	0.16	0.26	0.11	-0.05	-0.01	0.43*	0.25	0.2
Total *n*-9	0.33*	0.08	0.16	0.17	0.23	0.11	-0.05	0	0.43*	0.29	0.25
*n-6 PUFA*											
C18:2*n*-6 (LA)	-0.59	0.21	0.31	0.58**	-0.13	-0.01	0.11	0.12	0.34	0.65**	0.73**
C18:3*n*-6	0.33*	0.44**	0.1	0.72**	0.07	0.16	0.35*	0.14	0	0.24	0.40*
C20:3*n*-6	0.41**	0.47**	0.44**	0.86**	0.70**	0.44**	0.50**	0.39**	0.56**	0.65**	0.64**
C20:4*n*-6 (AA)	0.54**	0.54**	-0.21	0.54**	0.09	0.41**	0.41**	-0.15	0.38*	0.50**	0.12
C22:5*n-*6	0.52**	0.58**	0.60**	0.79**	0.76**	0.22	0.27	0.2	0.52**	0.68**	0.54**
Total *n*-6	-0.22	-0.07	-0.11	0.22	-0.04	-0.19	-0.25	-0.05	0.23	0.44*	0.22
*n-3 PUFA*											
C18:3*n*-3 (ALA)	0.17	0.06	0.07	0.70**	0.43**	0.33*	0.43**	0.22	0.31	0.1	-0.17
C20:4*n*-3 (ETA)	0.54**	0.59**	0.48**	0.92**	0.80**	0.36**	0.58**	0.48**	0.60**	0.59**	0.70**
C20:5*n*-3 (EPA)	0.81**	0.82**	0.62**	0.84**	0.72**	0.69**	0.72**	0.54**	0.84**	0.83**	0.83**
C22:5*n*-3 (DPA)	0.73**	0.67**	0.47**	0.83**	0.74**	0.55**	0.41**	0.49**	0.48**	0.51**	0.37*
C22:6*n*-3 (DHA)	0.68**	0.64**	0.59**	0.88**	0.80**	0.50**	0.59**	0.56**	0.61**	0.59**	0.64**
Total *n*-3	0.58**	0.61**	0.50**	0.85**	0.66**	0.45**	0.57**	0.55**	0.58**	0.64**	0.60**
*Sum*											
Σ SFA	-0.24	-0.25	0.05	0.52	-0.25	-0.05	-0.24	0.01	0.27	0.01	0.09
Σ MUFA	0.27	0.06	-0.06	0.12	0.14	0.00	0.05	-0.09	0.32	0.24	0.06
Σ PUFA	-0.21	-0.07	-0.2	0.23	-0.10	-0.16	-0.12	-0.01	0.17	0.09	0.11

^*‡*^Results of day 7 and day 56 of each intervention combined.

^1^*r* Pearson's correlation coefficient; * *P* ≤ 0.05, ** *P* ≤ 0.01.

For a higher power and better comparability of the correlation coefficients between baseline (n = 38) and the intervention period, the data at day 7 and day 56 of each treatment group were combined (test group n = 46, control group n = 30; Table 5). During the intervention with linseed oil and olive oil correlation coefficients of single FA of cheek cells to the respective FA in all blood fractions were similar to the baseline correlation coefficients. In general, the correlation of total *n*-3 PUFA and single *n*-3 PUFA between cheek cells and all blood fractions were always positive and significant which was independent of the treatment, except for ALA. The ALA intake by linseed oil resulted in significant correlation of ALA between cheek cells and plasma (*r* 0.33) or RBC (*r* 0.43) (Table 5), whereas without intervention and in the control group no correlations were found.

## Discussion

Since the 1980s, analyses of FA in cheek cells were undertaken as a non-invasive alternative to identify the dietary intake of FA [12,19]. However, only few studies evaluated cheek cell lipids as a marker for the FA status, especially for *n*-3 PUFA supply [10,11,20]–[24]. This study is the first which examined the changes in *n*-3 PUFA of cheek cells after intervention with ALA-rich linseed oil compared to a *n*-3 PUFA-free control oil. In addition, alterations in the FA composition of cheek cells were compared to three blood fractions.

Cheek cell sampling is a non-invasive method that can be easily performed in a non-clinical environment, and was tolerated well by all participants of the present study. Cheek cells were collected by rotary scraping with one brush at the inside of each cheek, followed by a mouth-rinse to collect remaining cells. This sampling procedure had been evaluated as the most effective adult sampling procedure by Klingler *et al.*[25] as well as by preliminary tests of our group. However, it has to be noted that the cell yield varied substantially between subjects. Previous data revealed a mean cell count of 3.5 ± 1.4 × 10^6^ cheek cells/per sampling [26]. Therefore, strict guidelines regarding the use of cheek cell lipids as markers for the FA status should be followed to avoid variation including sampling- and analytical procedures.

In general, PL are assumed to be the main component of cell membranes [21]. As lipids and especially PUFA are incorporated into cheek cell membranes, there is a focus on PL [20,21] and glycerophospholipids [11,25] in FA analysis. Advantageously, PL analysis of cheek cell FA is not much affected by food remains [21]. Our results confirmed PL as the major lipid class (57%) of cheek cells. However, regarding a higher lipid yield the separation of lipid classes was omitted in the present study and total lipids of cheek cells were used to analyze the FA composition as reported previously [19,23,24]. In order to identify possible food-remains in total lipids of cheek cells, a high amount of lauric acid (C12:0) is considered as marker for food contamination. Between 4-14% of lauric acid can be found in total FA of various diets, whereas cheek cells contain only <0.2% of this FA [20]. The mean portion of lauric acid in the present samples was 0.22% FAME (data not shown). Therefore, contamination via food remains could be excluded in our samples, as far as possible.

In the present study, the additional dietary intake of ~5 g/d ALA increased ALA portion of cheek cell lipids significantly (Table 3). Lapillone *et al.*, in their study on piglets [27] found similar results of cheek cell ALA after a linseed oil diet for two weeks. In human studies, the FA composition of cheek cells was previously only analyzed after the supplementation with DHA [11] or EPA + DHA [23] in adults, and *n*-3 PUFA-enriched formula in infants [10,20]. The present study demonstrated that cheek cells also represent endogenously synthesized FA. Besides the increase of the supplemented ALA, the long-chain *n*-3 PUFA metabolites such as ETA, EPA, and DPA also significantly increased in the cheek cell lipids after eight weeks of linseed oil intervention. Based on this study’s dietary *n*-3 PUFA restriction (no fish, fish oil, other *n*-3 PUFA-containing oils) these long-chain *n*-3 PUFA derived from the endogenous conversion of ALA. No changes occurred in DHA, which confirmed the very low conversion rate from ALA to DHA reported at <0.05% [28].

Cheek cells are known as a tissue with a fast regeneration time of approximately five days [13]. Therefore cheek cell FA are thought to be a biomarker detecting short-term changes in the diet, as it is known from plasma [6]. This assumption was confirmed by the present results. Successfully absorbed ALA was incorporated in cheek cell membranes and was detectable after already 7 days of a linseed oil intervention (Table 3). Moreover, after only one week of linseed oil free wash-out period (n = 7), ALA almost returned to its baseline values (day 0: 0.24; day 56: 0.48; wash-out day 63: 0.28% FAME).

Similarities were present when comparing measured cheek cell FA compositions at baseline with other studies, demonstrating the consistency of using cheek cells as tissue for FA analyses (Table 6). SFA, OA, and LA were confirmed as major FA while *n*-3 PUFA amounted for the smallest portion in previous studies, despite different analyzed lipid classes [11, 20; Staps and Kuhnt, unpublished] (Table 6). Notably, the cheek cell FA composition of the present study corresponded remarkably well to the study of Klingler *et al.*[11], especially regarding *n*-6 PUFA and *n-*3 PUFA.

**Table 6:** Comparison of FA composition and cheek cell correlation of the present study with relevant literature

	**Cheek cell FA composition**				**Cheek cell FA correlation**			
**Reference**	**Connor *et al. *** **(2000)**[20]	**Staps and Kuhnt (2010)[unpublished]**	**Klingler *et al. *** **(2013)**[11]	**Present study**	**Klingler *et al. *** **(2013)**[11]		**Present study**	
Study group	Control group	Total subjects; baseline values	Total subjects; baseline values	Total subjects; baseline values	Total subjects; baseline values		Total subjects; baseline values	
Subjects	Infants	Adults	Adults	Adults	Adults		Adults	
	(n = 8)	(n = 4)	(n = 13)	(n = 38)	(n = 13)		(n = 38)	
Lipid class	Phospholipids	Total lipids	Glycerophospholipids	Total lipids	Glycerophospholipids		Total lipids	
	*% FAME*	*% FAME*	*mol%*	*% FAME*	*r*^*1*^*Plasma*	*r*^*1*^*RBC*	*r*^*1*^*Plasma*	*r*^*1*^*RBC*
*SFA*								
C16:0	14.5	15.6	16.6	17.1	0.64*	0.32	-0.08	-0.10
C18:0	12.3	11.4	15.3	16.5	0.70**	0.33	0.04	-0.16
*n-9 MUFA*								
C18:1*n*-9 (OA)	25.8	28.9	30.1	26.7	0.40	0.10	0.34*	0.06
*n-6 PUFA*								
C18:2*n*-6 (LA)	16.5	18.4	17.3	17.1	-0.04	-0.05	-0.59	0.21
C20:4*n*-6 (AA)	2.00	4.37	3.20	3.32	0.65**	0.17	0.54***	0.54***
C22:5*n-*6	1.00	0.12	0.07	0.06	0.78***	0.70**	0.52***	0.58***
*n-3 PUFA*								
C18:3*n*-3 (ALA)	0.40	0.23	0.24	0.29	0.11	0.13	0.17	0.06
C20:5*n*-3 (EPA)	0.10	0.55	0.21	0.22	0.66	0.79**	0.81***	0.82***
C22:5*n*-3 (DPA)	0.20	0.43	0.23	0.22	0.85	0.39	0.73***	0.67***
C22:6*n*-3 (DHA)	0.40	1.07	0.69	0.79	0.76***	0.88***	0.68***	0.64***

^1^*r* Pearson's correlation coefficient; *P ≤ 0.05, **P ≤ 0.01, ***P ≤ 0.001.

In addition to cheek cell FA, the present study analyzed the FA of three blood fractions such as plasma, RBC and PBMC. Comparing the FA profile of these blood fractions with the cheek cell FA profile, strong baseline variations were found (Table 4). After intervention with either linseed oil or olive oil, similar changes occurred in blood matrices (data not shown) as well as in cheek cell FA (Table 3). Interestingly, the increase of the precursor ALA in the blood fractions (~320%) was higher compared to the increase of ALA in cheek cells after linseed oil intervention (87%), while the increase of the metabolite EPA was similar (47% vs. 56%). Thus, cheek cells lipids reflect dietary FA, but also endogenous metabolism. The supplementation of linseed oil led to an enrichment of *n*-3 PUFA in all four analyzed materials and thus decreased the ratios of *n*-6/*n*-3 PUFA and AA/EPA. Therefore, linseed oil consumption could contribute to beneficial health effects such as a reduction of the CVD risk by improving the *n*-3 PUFA status in the body [1]. A general use of linseed oil as a replacement for the commonly used high *n*-6 PUFA plant-oils of the Western diet can be recommended to improve the *n*-6/*n-*3 PUFA ratio in the diet.

In general, the standard analysis of the FA status and the *n*-3 PUFA supply in humans is mostly conducted by analyzing the FA of plasma, RBC, platelets or adipose tissue as they strongly correlate with the FA content of the diet [8]. Until now, only little was known about the correlation of dietary FA intake and cheek cell FA composition. However, in some previous studies cheek cell FA composition was used as marker for the FA supply and correlations with plasma- and RBC FA were detected [10,11,20,21]. Cheek cells were also reported to reflect the nutritional intake of FA [10]. Thus, high correlations were found between dietary AA and cheek cell AA (*r* 0.51) as well as between dietary DHA and cheek cell DHA (*r* 0.65) [20]. The present study did not analyze the correlation between diet and cheek cell FA, but showed strong correlations of cheek cell FA with the respective FA of plasma and RBC, which are both known markers of FA intake. This emphasizes cheek cell FA as a biomarker for the FA supply of the diet. As this was the first study to analyze the correlation between cheek cell FA and PBMC FA to this extent, it can be noted that less correlations exist compared to the correlation analyses between cheek cells and plasma or RBC. However, in cases of *n*-3 PUFA the correlations between cheek cells and PBMC are still highly significant (*r* 0.47–0.62; Table 5).

Upon closer examination high correlations of cheek cell EPA and DHA to the respective FA of plasma and RBC were previously described [11,20,25,27]. This could be confirmed by the present results regarding the correlations of *n*-3 PUFA ETA, EPA, DPA, DHA, and total *n*-3 PUFA between cheek cells and plasma, RBC as well as PBMC (Table 5). Otherwise, cheek cell FA seemed to be unsuitable as an adequate biomarker for SFA in body lipids shown by the present results and Skeaff *et al.*[29]. Paradoxically, total *n*-6 PUFA of cheek cells correlated negatively to the total *n*-6 PUFA of the analyzed blood lipids despite major part of individual *n*-6 PUFA correlated positively (Table 5). Laitinen *et al.* found similar observations with regard to correlations of *n*-6 PUFA between cheek cell- and serum FA [22]. Most likely this could be due to the different amounts and opposed portions of LA and AA seen in the various blood fractions (Table 4). However, best correlations were found between plasma and RBC.

When comparing the correlation analysis by Klingler *et al.*[11] with the present results at baseline, similar results were obtained indicating again the consistency of this method (Table 6). No correlations were found for the essential FA LA and ALA between cheek cells and plasma or RBC in both studies. Despite a lower subject number in the study of Klingler *et al.*[11], correlations between cheek cells and plasma or RBC in AA, DPA*n*-6, EPA, and DHA were reported. Especially significant correlation of EPA and DHA of cheek cells with plasma and RBC (*r* 0.61-0.94) were also observed in the other studies [20,25,27].

Based on the present knowledge, cheek cell FA mainly represented *n*-3 PUFA and *n-*6 PUFA status related to plasma and RBC. However, this did not apply for SFA. Especially when blood samples are not available, cheek cell lipids could represent an alternative.

## Conclusions

The present results indicate cheek cell FA as a non-invasive biomarker for both dietary *n-*3 PUFA supply and endogenously derived *n-*3 PUFA metabolites. The present results indicated that ALA intake by linseed oil increased EPA and lowered the *n*-6/*n*-3 PUFA ratio in cheek cell lipids. Cheek cell FA can used as alternative to FA of plasma and RBC to determine dietary FA intake, with special regard to *n*-3 PUFA. In general, the FA profile of cheek cells mainly reflected short-term changes. Furthermore, the non-invasive sampling procedure is an advantage to study participants and can be performed independently of clinical personnel. Therefore, the assessment of cheek cell FA can be considered for human intervention studies and large-scale epidemiological studies where the *n*-3 PUFA supply and its metabolism are of interest.

## Abbreviations

AA: Arachidonic acid; ALA: Alpha-linolenic acid; BMI: Body mass index; CVD: Cardiovascular diseases; DHA: Docosahexaenoic acid; DPA: Docosapentaenoic acid; EPA: Eicosapentaenoic acid; ETA: Eicosatetraenoic acid; FA: Fatty acid; FAME: Fatty acid methyl esters; LA: Linoleic acid; MUFA: Monounsaturated fatty acids; n-3: Omega-3; PUFA: Polyunsaturated fatty acids; OA: Oleic acid; PBMC: Peripheral blood mononuclear cells; PL: Phospholipids; RBC: Red blood cells; SD: Standard deviation; SFA: Saturated fatty acids.

## Competing interests

The authors declare that they have no competing interests.

## Authors' contributions

KK was investigator of the study. AG and FS participated in the study implementation, carried out cheek cell sampling and fatty acid analyses. AG and KK carried out the statistical analyses and draft the manuscript. All authors have read and approved the final manuscript.
